# *N*-(4-Methyl­phenyl­sulfon­yl)succinamic acid

**DOI:** 10.1107/S1600536812023276

**Published:** 2012-05-26

**Authors:** H. Purandara, Sabine Foro, B. Thimme Gowda

**Affiliations:** a Department of Chemistry, Mangalore University, Mangalagangotri 574 199, Mangalore, India; b Institute of Materials Science, Darmstadt University of Technology, Petersenstrasse 23, D-64287 Darmstadt, Germany

## Abstract

In the crystal structure of the title compound, C_11_H_13_NO_5_S, the amide C=O and the carboxyl C=O groups of the acid segment orient themselves away from each other. The dihedral angle between the benzene ring and the amide group is 69.0 (2)°. In the crystal, N—H⋯O and O—H⋯O hydrogen bonds link the mol­ecules into layers parallel to the *bc* plane.

## Related literature

For our studies on the effects of substituents on the structures and other aspects of *N*-(ar­yl)-amides, see: Gowda *et al.* (2000 ▶); Saraswathi *et al.* (2011 ▶), of *N*-chloro­aryl­amides, see: Gowda & Rao (1989 ▶); Jyothi & Gowda (2004 ▶) and of *N*-bromo­aryl­sulfonamides, see: Gowda & Mahadevappa (1983 ▶); Usha & Gowda (2006 ▶).
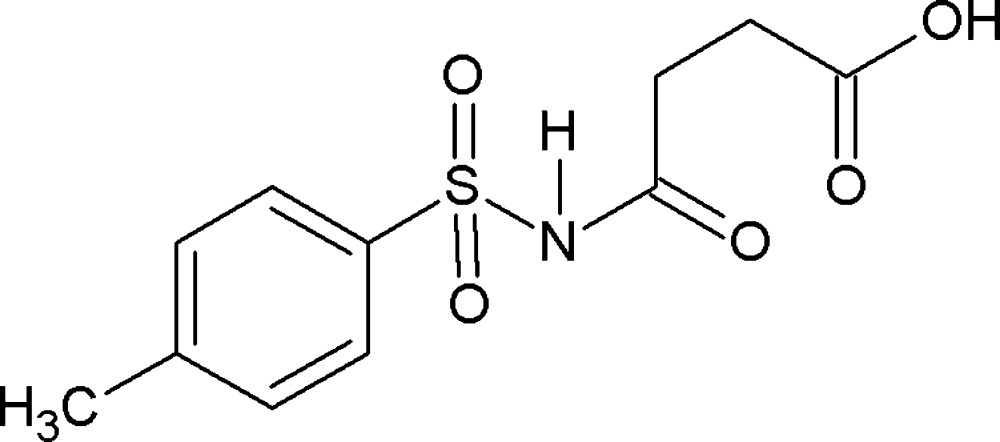


## Experimental

### 

#### Crystal data

C_11_H_13_NO_5_S
*M* *_r_* = 271.28
Monoclinic, 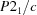

*a* = 10.2496 (9) Å
*b* = 17.041 (2) Å
*c* = 7.4721 (6) Å
β = 101.909 (9)°
*V* = 1277.0 (2) Å^3^
*Z* = 4
Mo *K*α radiation
μ = 0.27 mm^−1^
*T* = 293 K
0.48 × 0.32 × 0.16 mm

#### Data collection

Oxford Diffraction Xcalibur diffractometer with a Sapphire CCD detector
Absorption correction: multi-scan (*CrysAlis RED*; Oxford Diffraction, 2009 ▶) *T*_min_ = 0.883, *T*_max_ = 0.959
4739 measured reflections
2597 independent reflections
2107 reflections with *I* > 2σ(*I*)
*R*_int_ = 0.014

#### Refinement

*R*[*F* ^2^ > 2σ(*F* ^2^)] = 0.046
*wR*(*F* ^2^) = 0.110
*S* = 1.12
2597 reflections
170 parameters
2 restraints
H atoms treated by a mixture of independent and constrained refinement
Δρ_max_ = 0.23 e Å^−3^
Δρ_min_ = −0.32 e Å^−3^

### 

Data collection: *CrysAlis CCD* (Oxford Diffraction, 2009 ▶); cell refinement: *CrysAlis RED* (Oxford Diffraction, 2009 ▶); data reduction: *CrysAlis RED*; program(s) used to solve structure: *SHELXS97* (Sheldrick, 2008 ▶); program(s) used to refine structure: *SHELXL97* (Sheldrick, 2008 ▶); molecular graphics: *PLATON* (Spek, 2009 ▶); software used to prepare material for publication: *SHELXL97*.

## Supplementary Material

Crystal structure: contains datablock(s) I, global. DOI: 10.1107/S1600536812023276/rz2761sup1.cif

Structure factors: contains datablock(s) I. DOI: 10.1107/S1600536812023276/rz2761Isup2.hkl

Supplementary material file. DOI: 10.1107/S1600536812023276/rz2761Isup3.cml

Additional supplementary materials:  crystallographic information; 3D view; checkCIF report

## Figures and Tables

**Table 1:** Hydrogen-bond geometry (Å, °)

*D*—H⋯*A*	*D*—H	H⋯*A*	*D*⋯*A*	*D*—H⋯*A*
N1—H1*N*⋯O1^i^	0.83 (2)	2.14 (2)	2.948 (2)	164 (2)
O5—H5*O*⋯O4^ii^	0.83 (2)	1.83 (2)	2.663 (3)	178 (3)

Symmetry codes: (i) 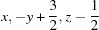; (ii) 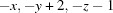.

**Fig. 1.**
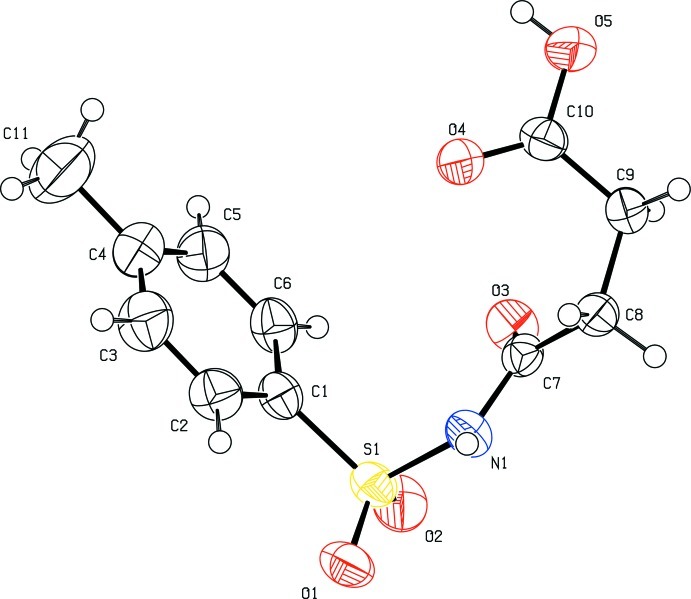
The molecular structure of the title compound, showing displacement ellipsoids drawn at the 50% probability level.

**Fig. 2.**
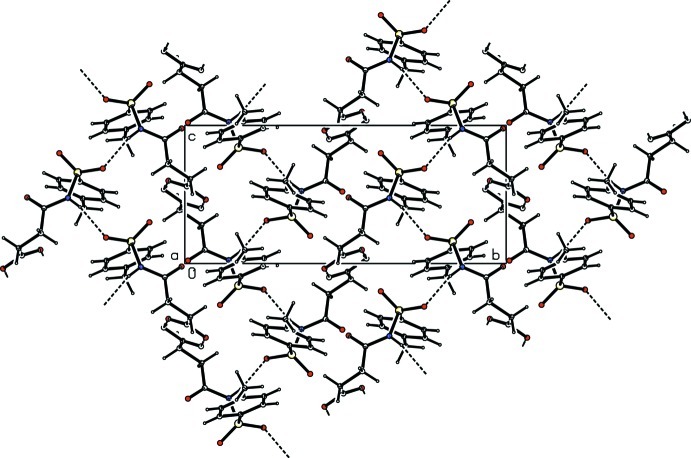
The molecular packing of the title compound with hydrogen bonding shown as dashed lines.

## supplementary crystallographic information

### Comment

As part of our studies on the substituent effects on the structures and other aspects of *N*-(aryl)-amides (Gowda *et al.*, 2000; Saraswathi *et al.*, 2011); *N*-chloroarylsulfonamides (Gowda & Rao, 1989; Jyothi & Gowda, 2004) and *N*-bromoaryl- sulfonamides (Gowda & Mahadevappa, 1983; Usha & Gowda, 2006), in the present work, the crystal structure of *N*-(4-methylphenylsulfonyl)succinamic acid has been determined (Fig. 1). The conformations of the N—H and C=O bonds in the amide segment are *anti* to each other. Further, the amide C═O and the carboxyl C═O of the acid segment orient themselves away from each other, in contrast to the *anti* conformation observed between the the amide oxygen and the carboxyl oxygen in *N*-(4-methylphenyl)-succinamic acid (I) (Saraswathi *et al.*, 2011). But both the amide oxygen and the carboxyl oxygen are *anti* to the H atoms on the adjacent –CH_2_ groups, in both the compounds.

In the title compound, the C═O and O—H bonds of the acid group are in *syn* position to each other, similar to that observed in (I). The molecule is bent at the S-atom with the C1–S1–N1–C7 torsion angle of 79.2 (1)°. Further, the dihedral angle between the phenyl ring and the amide group is 69.0 (2)°. In the crystal, the pairs of O—H···O and N—H···O intermolecular hydrogen bonds link the molecules into layers parallel to the *bc* plane (Table 1, Fig. 2).

### Experimental

Succinic anhydride (0.015 mole) and 4-dimethylaminopyridine (0.01 mole) were added to a solution of *p*-toluenesulfonamide (0.01 mole) in dichloromethane. The reaction mixture was strirred for 18 h at room temperature and set aside for completion of the reaction. The reaction mixture was concentrated to dryness. The resultant title compound was washed with dilute HCl and then with water thoroughly, to remove the unreacted base and the succinic anhydride. It was recrystallized to constant melting point from ethyl acetate (173–175 °C). The purity of the compound was checked and characterized by its infrared spectrum. Prism-like colourless single crystals used in X-ray diffraction studies were grown by slow evaporation of an ethyl acetate solution at room temperature.

### Refinement

The H atoms of the NH group and the OH group were located in a difference Fourier map and later restrained to the distances of N—H = 0.86 (2) Å and O—H = 0.82 (2) Å, respectively. The other H atoms were positioned with idealized geometry using a riding model with the aromatic C—H = 0.93 Å, methyl C—H = 0.96 Å and methylene C—H = 0.97 Å. All H atoms were refined with isotropic displacement parameters set at 1.2 *U*_eq_(C-aromatic, N) and 1.5 *U*_eq_(*C*-methyl).

### Crystal data

**Table:**

C_11_H_13_NO_5_S	*F*(000) = 568
*M**_r_* = 271.28	*D*_x_ = 1.411 Mg m^−^^3^
Monoclinic, *P*2_1_/*c*	Mo *K*α radiation, λ = 0.71073 Å
Hall symbol: -P 2ybc	Cell parameters from 2495 reflections
*a* = 10.2496 (9) Å	θ = 3.0–27.6°
*b* = 17.041 (2) Å	µ = 0.27 mm^−^^1^
*c* = 7.4721 (6) Å	*T* = 293 K
β = 101.909 (9)°	Prism, colourless
*V* = 1277.0 (2) Å^3^	0.48 × 0.32 × 0.16 mm
*Z* = 4	

### Data collection

**Table:**

Oxford Diffraction Xcalibur diffractometer with a Sapphire CCD detector	2597 independent reflections
Radiation source: fine-focus sealed tube	2107 reflections with *I* > 2σ(*I*)
Graphite monochromator	*R*_int_ = 0.014
Rotation method data acquisition using ω and phi scans	θ_max_ = 26.4°, θ_min_ = 3.0°
Absorption correction: multi-scan (*CrysAlis RED*; Oxford Diffraction, 2009)	*h* = −12→11
*T*_min_ = 0.883, *T*_max_ = 0.959	*k* = −21→8
4739 measured reflections	*l* = −9→9

### Refinement

**Table:**

Refinement on *F*^2^	Primary atom site location: structure-invariant direct methods
Least-squares matrix: full	Secondary atom site location: difference Fourier map
*R*[*F*^2^ > 2σ(*F*^2^)] = 0.046	Hydrogen site location: inferred from neighbouring sites
*wR*(*F*^2^) = 0.110	H atoms treated by a mixture of independent and constrained refinement
*S* = 1.12	*w* = 1/[σ^2^(*F*_o_^2^) + (0.034*P*)^2^ + 0.9166*P*] where *P* = (*F*_o_^2^ + 2*F*_c_^2^)/3
2597 reflections	(Δ/σ)_max_ = 0.001
170 parameters	Δρ_max_ = 0.23 e Å^−^^3^
2 restraints	Δρ_min_ = −0.32 e Å^−^^3^

### Special details

**Table:**

Experimental. Absorption correction: CrysAlis RED (Oxford Diffraction, 2009) Empirical absorption correction using spherical harmonics, implemented in SCALE3 ABSPACK scaling algorithm.
Geometry. All e.s.d.'s (except the e.s.d. in the dihedral angle between two l.s. planes) are estimated using the full covariance matrix. The cell e.s.d.'s are taken into account individually in the estimation of e.s.d.'s in distances, angles and torsion angles; correlations between e.s.d.'s in cell parameters are only used when they are defined by crystal symmetry. An approximate (isotropic) treatment of cell e.s.d.'s is used for estimating e.s.d.'s involving l.s. planes.
Refinement. Refinement of *F*^2^ against ALL reflections. The weighted *R*-factor *wR* and goodness of fit *S* are based on *F*^2^, conventional *R*-factors *R* are based on *F*, with *F* set to zero for negative *F*^2^. The threshold expression of *F*^2^ > σ(*F*^2^) is used only for calculating *R*-factors(gt) *etc*. and is not relevant to the choice of reflections for refinement. *R*-factors based on *F*^2^ are statistically about twice as large as those based on *F*, and *R*- factors based on ALL data will be even larger.

### Fractional atomic coordinates and isotropic or equivalent isotropic displacement parameters (Å^2^)

**Table:**

	*x*	*y*	*z*	*U*_iso_*/*U*_eq_	
C1	0.1649 (2)	0.82620 (14)	0.0924 (3)	0.0398 (5)	
C2	0.1077 (3)	0.75638 (15)	0.0207 (4)	0.0507 (6)	
H2	0.1604	0.7126	0.0132	0.061*	
C3	−0.0291 (3)	0.75296 (17)	−0.0394 (4)	0.0625 (7)	
H3	−0.0682	0.7063	−0.0881	0.075*	
C4	−0.1092 (3)	0.81770 (18)	−0.0286 (4)	0.0607 (7)	
C5	−0.0496 (3)	0.88660 (16)	0.0469 (4)	0.0582 (7)	
H5	−0.1025	0.9301	0.0572	0.070*	
C6	0.0864 (3)	0.89152 (14)	0.1068 (4)	0.0482 (6)	
H6	0.1255	0.9381	0.1563	0.058*	
C7	0.3668 (2)	0.93791 (13)	−0.0993 (3)	0.0349 (5)	
C8	0.4086 (2)	0.94773 (14)	−0.2799 (3)	0.0394 (5)	
H8A	0.3879	0.9001	−0.3514	0.047*	
H8B	0.5042	0.9557	−0.2582	0.047*	
C9	0.3388 (2)	1.01660 (14)	−0.3874 (3)	0.0439 (6)	
H9A	0.3582	1.0638	−0.3143	0.053*	
H9B	0.3743	1.0236	−0.4971	0.053*	
C10	0.1912 (2)	1.00645 (14)	−0.4408 (3)	0.0419 (5)	
C11	−0.2586 (3)	0.8130 (2)	−0.0975 (6)	0.0995 (13)	
H11A	−0.2856	0.7590	−0.1091	0.119*	
H11B	−0.2822	0.8382	−0.2147	0.119*	
H11C	−0.3029	0.8389	−0.0126	0.119*	
N1	0.3902 (2)	0.86326 (11)	−0.0248 (2)	0.0387 (4)	
H1N	0.404 (3)	0.8261 (12)	−0.090 (3)	0.046*	
O1	0.39301 (18)	0.75502 (11)	0.1880 (2)	0.0565 (5)	
O2	0.37311 (18)	0.89014 (11)	0.2992 (2)	0.0558 (5)	
O3	0.32175 (17)	0.99012 (10)	−0.0214 (2)	0.0479 (4)	
O4	0.13237 (16)	0.94917 (10)	−0.4003 (3)	0.0552 (5)	
O5	0.13192 (19)	1.06525 (12)	−0.5367 (3)	0.0672 (6)	
H5O	0.0496 (18)	1.0596 (19)	−0.557 (4)	0.081*	
S1	0.33860 (6)	0.83267 (4)	0.15920 (7)	0.04120 (18)	

### Atomic displacement parameters (Å^2^)

**Table:**

	*U*^11^	*U*^22^	*U*^33^	*U*^12^	*U*^13^	*U*^23^
C1	0.0465 (13)	0.0405 (13)	0.0342 (11)	−0.0018 (10)	0.0130 (9)	0.0004 (10)
C2	0.0567 (15)	0.0388 (13)	0.0565 (15)	0.0028 (12)	0.0116 (12)	−0.0042 (12)
C3	0.0639 (18)	0.0487 (16)	0.0723 (19)	−0.0113 (14)	0.0080 (14)	−0.0089 (14)
C4	0.0482 (15)	0.0634 (18)	0.0701 (18)	−0.0024 (13)	0.0112 (13)	0.0047 (15)
C5	0.0537 (16)	0.0479 (15)	0.0771 (19)	0.0076 (13)	0.0230 (14)	0.0025 (14)
C6	0.0562 (15)	0.0359 (13)	0.0564 (15)	−0.0021 (11)	0.0209 (12)	−0.0030 (11)
C7	0.0315 (11)	0.0392 (12)	0.0328 (11)	−0.0044 (9)	0.0038 (9)	−0.0022 (9)
C8	0.0365 (12)	0.0479 (13)	0.0336 (11)	−0.0005 (10)	0.0072 (9)	0.0029 (10)
C9	0.0405 (12)	0.0479 (14)	0.0430 (12)	−0.0051 (11)	0.0082 (10)	0.0080 (11)
C10	0.0464 (13)	0.0400 (13)	0.0376 (12)	0.0007 (11)	0.0047 (10)	0.0042 (10)
C11	0.054 (2)	0.098 (3)	0.140 (4)	−0.0050 (19)	0.003 (2)	−0.001 (3)
N1	0.0485 (11)	0.0360 (10)	0.0341 (10)	0.0027 (9)	0.0142 (8)	−0.0011 (8)
O1	0.0609 (11)	0.0531 (11)	0.0559 (11)	0.0110 (9)	0.0129 (9)	0.0202 (9)
O2	0.0649 (11)	0.0689 (12)	0.0326 (8)	−0.0113 (10)	0.0075 (8)	−0.0062 (8)
O3	0.0580 (10)	0.0423 (10)	0.0467 (9)	0.0046 (8)	0.0181 (8)	−0.0041 (8)
O4	0.0420 (9)	0.0466 (10)	0.0718 (12)	−0.0054 (8)	−0.0001 (8)	0.0162 (9)
O5	0.0451 (10)	0.0579 (12)	0.0924 (15)	0.0011 (9)	0.0001 (10)	0.0301 (11)
S1	0.0478 (3)	0.0445 (3)	0.0315 (3)	−0.0001 (3)	0.0087 (2)	0.0046 (2)

### Geometric parameters (Å, º)

**Table:**

C1—C2	1.385 (3)	C8—H8A	0.9700
C1—C6	1.390 (3)	C8—H8B	0.9700
C1—S1	1.750 (2)	C9—C10	1.493 (3)
C2—C3	1.382 (4)	C9—H9A	0.9700
C2—H2	0.9300	C9—H9B	0.9700
C3—C4	1.387 (4)	C10—O4	1.218 (3)
C3—H3	0.9300	C10—O5	1.307 (3)
C4—C5	1.388 (4)	C11—H11A	0.9600
C4—C11	1.514 (4)	C11—H11B	0.9600
C5—C6	1.377 (4)	C11—H11C	0.9600
C5—H5	0.9300	N1—S1	1.6559 (19)
C6—H6	0.9300	N1—H1N	0.828 (16)
C7—O3	1.206 (3)	O1—S1	1.4349 (19)
C7—N1	1.390 (3)	O2—S1	1.4234 (18)
C7—C8	1.507 (3)	O5—H5O	0.832 (18)
C8—C9	1.516 (3)		
			
C2—C1—C6	120.8 (2)	H8A—C8—H8B	107.9
C2—C1—S1	119.28 (19)	C10—C9—C8	113.14 (19)
C6—C1—S1	119.88 (19)	C10—C9—H9A	109.0
C3—C2—C1	118.8 (2)	C8—C9—H9A	109.0
C3—C2—H2	120.6	C10—C9—H9B	109.0
C1—C2—H2	120.6	C8—C9—H9B	109.0
C2—C3—C4	121.3 (3)	H9A—C9—H9B	107.8
C2—C3—H3	119.3	O4—C10—O5	123.6 (2)
C4—C3—H3	119.3	O4—C10—C9	123.6 (2)
C3—C4—C5	118.7 (3)	O5—C10—C9	112.9 (2)
C3—C4—C11	120.5 (3)	C4—C11—H11A	109.5
C5—C4—C11	120.8 (3)	C4—C11—H11B	109.5
C6—C5—C4	121.0 (2)	H11A—C11—H11B	109.5
C6—C5—H5	119.5	C4—C11—H11C	109.5
C4—C5—H5	119.5	H11A—C11—H11C	109.5
C5—C6—C1	119.3 (2)	H11B—C11—H11C	109.5
C5—C6—H6	120.3	C7—N1—S1	124.26 (16)
C1—C6—H6	120.3	C7—N1—H1N	120.1 (18)
O3—C7—N1	122.2 (2)	S1—N1—H1N	111.6 (18)
O3—C7—C8	123.9 (2)	C10—O5—H5O	111 (2)
N1—C7—C8	113.78 (19)	O2—S1—O1	119.62 (11)
C7—C8—C9	111.76 (19)	O2—S1—N1	108.68 (10)
C7—C8—H8A	109.3	O1—S1—N1	103.54 (10)
C9—C8—H8A	109.3	O2—S1—C1	109.68 (11)
C7—C8—H8B	109.3	O1—S1—C1	109.01 (11)
C9—C8—H8B	109.3	N1—S1—C1	105.27 (10)
			
C6—C1—C2—C3	1.0 (4)	C8—C9—C10—O4	0.8 (3)
S1—C1—C2—C3	−177.0 (2)	C8—C9—C10—O5	−178.6 (2)
C1—C2—C3—C4	−0.2 (4)	O3—C7—N1—S1	10.0 (3)
C2—C3—C4—C5	−0.9 (5)	C8—C7—N1—S1	−172.55 (15)
C2—C3—C4—C11	179.2 (3)	C7—N1—S1—O2	−48.4 (2)
C3—C4—C5—C6	1.3 (4)	C7—N1—S1—O1	−176.60 (18)
C11—C4—C5—C6	−178.8 (3)	C7—N1—S1—C1	69.0 (2)
C4—C5—C6—C1	−0.5 (4)	C2—C1—S1—O2	−153.06 (19)
C2—C1—C6—C5	−0.7 (4)	C6—C1—S1—O2	28.8 (2)
S1—C1—C6—C5	177.4 (2)	C2—C1—S1—O1	−20.3 (2)
O3—C7—C8—C9	−23.2 (3)	C6—C1—S1—O1	161.55 (18)
N1—C7—C8—C9	159.36 (19)	C2—C1—S1—N1	90.2 (2)
C7—C8—C9—C10	−63.7 (3)	C6—C1—S1—N1	−87.9 (2)

### Hydrogen-bond geometry (Å, º)

**Table:**

*D*—H···*A*	*D*—H	H···*A*	*D*···*A*	*D*—H···*A*
N1—H1*N*···O1^i^	0.83 (2)	2.14 (2)	2.948 (2)	164 (2)
O5—H5*O*···O4^ii^	0.83 (2)	1.83 (2)	2.663 (3)	178 (3)

Symmetry codes: (i) *x*, −*y*+3/2, *z*−1/2; (ii) −*x*, −*y*+2, −*z*−1.
